# Significant decline of HPV 6 infection and genital warts despite low HPV vaccination coverage in young women in Germany: a long-term prospective, cohort data analysis

**DOI:** 10.1186/s12879-021-06139-y

**Published:** 2021-07-02

**Authors:** Agnieszka Denecke, Thomas Iftner, Angelika Iftner, Sebastian Riedle, Marion Ocak, Alexander Luyten, Isak Üye, Kübra Tunc, Karl Ulrich Petry

**Affiliations:** 1 Department of Obstetrics and Gynecology, Klinikum Wolfsburg, Wolfsburg, Germany; 2 Department of Obstetrics, Gynecology and Reproductive Medicine, Medical Hannover School, Hannover, Germany; 3 Institute of Medical Virology, University of Tübingen, Tübingen, Germany; 4 MD research, Statistics in clinical research, Pullach i, Isartal, Germany; 5 Abts+partner, Frauenärzte, Prüner Gang, Kiel, Germany

**Keywords:** Human papillomavirus, Genital warts, Condylomata acuminata, HPV-vaccination

## Abstract

**Background:**

The introduction of human papillomavirus (HPV) vaccination has resulted in a remarkable decline of genital warts in women and men, but in Germany historical rates of vaccination are relatively low. We report long-term surveillance data on changes in HPV 6 and HPV 11 infection and the prevalence of genital warts in young women in the Wolfsburg HPV epidemiological study (WOLVES).

**Methods:**

Women born in 1983/84, 1988/89, and 1993/94 participated in four cohorts between 2009/10 and 2014/15. Quadrivalent vaccination coverage and prevalence of HPV 6/11 infection and genital warts are reported for participants aged 19–22 years and 24–27 years at the time of sample collection. Statistical analyses were done to compare similarly aged participants using 2 × 2 contingency tables (Röhmel-Mansmann unconditional exact test; two-side alpha of 0.05).

**Results:**

A total of 2456 women were recruited. Between 2010 and 2015, there was a statistically significant decrease in the prevalence of HPV 6 infection among women aged 24–27 years (2.1% versus 0.0%; *P* < 0.0001) and women aged 19–22 years (2.0% versus 0.0%; *P* = 0.0056). There was no significant decline in HPV 11 infection. In total, 52 of 2341 participants were diagnosed with genital warts. There was a statistically significant drop in the risk of developing genital warts in women aged 24–27 years between 2010 and 2015 (4.7% versus 1.7%, respectively; *P* = 0.0018). The overall risk of developing genital warts in women aged 19–27 years decreased from 3.1% in 2010 to 1.2% in 2015 (*P* = 0.0022).

**Conclusions:**

An increase in vaccination coverage was associated with a decreased prevalence of genital warts in young women. A protective effect greater than herd immunity alone was seen despite low vaccination rates. Quadrivalent vaccine had a protective effect on genital HPV 6 infection and an almost fully protective effect on the development of genital warts in the youngest population.

## Background

Human papillomavirus (HPV) is one of the most frequently sexually transmitted viral infections in the world [1]. Persistent infections with high-risk HPV types (or class I and IIa carcinogenic types as defined by the International Agency for Research on Cancer) is a necessary risk factor for the development of cervical cancer, other anogenital malignancies, oropharyngeal cancer, and possibly nonmelanoma skin tumors, while low-risk and other HPV types can lead to benign tumors of the skin and mucosa, for example, genital warts. The term ‘genital wart’ is not well-defined and covers a wide spectrum of diverse skin and mucosal lesions including the typical condylomata acuminata [2], associated with infection with low-risk HPV genotypes 6 and 11 [3]. Risk factors include acquisition of new sex partners, a higher number of sex partners, and concurrent infection with high-risk HPV types [4]. Condylomata acuminata are highly contagious and will develop in approximately 65% of individuals with an infected partner [5].

Prophylactic HPV vaccines, either the quadrivalent vaccine against HPV types 6, 11 and 16, 18 or the nonavalent vaccine against types 6, 11, 16, 18, 31, 33, 45, 52, and 58 can minimize the overall burden of HPV and genital warts [6]. An Australian vaccination program using the quadrivalent HPV vaccine quickly led to a decline and almost complete disappearance of genital warts in both women and men [7]. The steep decrease in the prevalence of genital warts occurred after the initiation of routine vaccination for girls but before widespread HPV immunization started in boys in 2013, which suggests that herd immunity protected the nonvaccinated male population [7, 8].

In contrast to countries with school-based HPV vaccination programs, such as Australia, the UK, and Austria, Germany has a relatively low vaccine coverage [9]. Although HPV vaccination for the prevention of cervical cancer was recommended in March 2007 for females aged 12–17 years, in 2008–2009 the vaccine uptake rate was about 40% in females aged 16–18 years and remained at a low 42% vaccination rate in 2017. Genital warts and HPV infections are not reportable diseases and reliable surveillance data are not available in Germany to estimate the incidence of genital warts and the impact of HPV vaccination [10]. Additional data from surveillance studies are necessary to understand the impact of HPV vaccination on the prevalence of HPV infection and associated genital warts. Here we report long-term surveillance data on changes in the incidence and prevalence of HPV 6 and 11 infections and the incidence and prevalence of genital warts observed between 2009/10 and 2014/15 in women, aged 19–22 years and 24–27 years, participating in the Wolfsburg HPV epidemiological study (WOLVES). The trends in the development of high-risk HPV infection are not part of this paper.

## Methods

The original design of the WOLVES study in the pre-vaccination era has been described previously [2]. The ongoing study is a long-term, population-based, prospective, cohort study to measure changes in the prevalence of HPV infections and associated diseases after the introduction of HPV vaccines among the general female population from 2009 to 2020. Voluntary participation in the WOLVES study was open to women irrespective of existing HPV status and all participants gave written informed consent. The study was approved by the local ethics committee (Bo/07/2009).

**Fig. 1:**
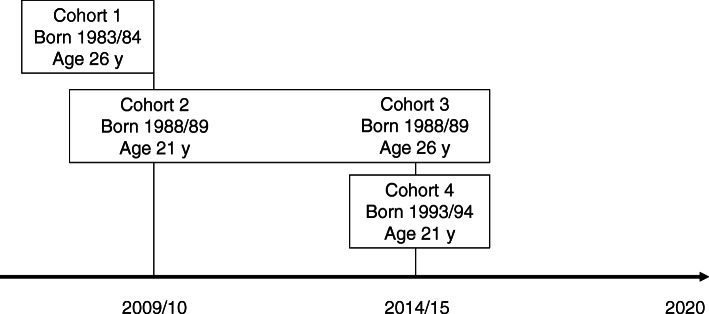
Timeline for recuitment and analysis of particpants in the study. Cohort 1 = born 1983/84 analyzed in 2009/10; cohort 2 = born in 1988/89 analyzed in 2009/10; cohort 3 = born 1988/89 analyzed in 2014/15; cohort 4 = born 1993/94 analyzed in 2014/15

Four cohorts are reported in this analysis: cohort 1 = participants born in 1983/84 and analyzed in 2009/10; cohort 2 = participants born in 1988/89 and analyzed in 2009/10; cohort 3 = participants born in 1988/89 and analyzed in 2014/15; and cohort 4 = participants born in 1993/94 and analyzed in 2014/15 (Fig. 1). At the time of sample collection, participants were aged 19–22 years in cohorts 2 and 4, and aged 24–27 years in cohorts 1 and 3. Women born in cohort 1 had a single cross-sectional medical examination in 2009/10, whereas those in cohorts 2 and 3 had annual medical examinations from 2009/10 to 2014/15. Participants in cohort 4 were analyzed in 2014/15 and will have annual visits until the end of 2020.

Participants of WOLVES do not differ from the general population of the same age, living in Wolfsburg area city, in terms of education, migration background, or parity. Town registry does not include any medical information, which is the main limitation of this study.

All participants were asked to complete a standardized medical questionnaire and attend regular medical examinations, as described previously [2]. The gynecologist collected data on HPV vaccination status by checking the certificate of vaccination, which includes all vaccinations. For HPV vaccinations, the certificate lists batch numbers and the date of vaccination. A fully vaccinated status, based on number of doses administered according to the recommended schedule, was defined as three doses given at months 0, 2, and 6 (5–13 months).

Women were referred to colposcopy if they had genital warts or an abnormal high-grade Pap smear, or they had an abnormal Pap smear classified as borderline or low-grade and tested positive for high-risk HPV infection. All colposcopy examinations were done at the Klinikum Wolfsburg, the single certified colposcopy unit in the region.

Women were not included if they were not living in the Wolfsburg area, had a diagnosis of cervical or genital cancer, another malignancy, an organ transplant, or were undergoing an immunosuppressive therapy.

Diagnosis of genital warts was based on colposcopy and histological biopsies. The analysis was based on the diagnosis of genital warts and also includes data on untreated genital warts. Genital warts were classified as: (i) typical condylomata acuminata for lesions with typical acuminate morphology, with typical punctuated or cauliflower-like patterns and tend to be pigmentless and are mostly seen on pigmented skin [11]; (ii) flat genital condylomata with a more hyperkeratotic and pigmented surface and flat condylomata with a smooth surface and non-pigmented papules; or (iii) seborrheic wart-like lesions of the cutaneous skin of the external anogenital area. The focus of this paper is on low-risk HPV types and associated disease therefore we do not report on vulvar intraepithelial neoplasia, Bowenoid papulosis, Naevi or Mollusca contagiosa [2]. These patients were excluded from the analysis of genital warts.

### HPV DNA testing

HPV testing and genotyping were done using the Hybrid Capture 2 assay (HC2; Qiagen Inc., Hilden Germany) and SPF-10-PCR followed by Reverse Line Probe Assay LiPA Extra, respectively, as described in detail previously [2, 12, 13]. Cervical Pap smear samples were analyzed for the presence of the 13 high-risk HPV types (16, 18, 31, 33, 35, 39, 45, 51, 52, 56, 58, 59, and 68) and the five low-risk HPV types (6, 11, 42, 43, and 44). Samples were diagnosed as positive if they attained or exceeded the FDA-approved threshold [2]. The INNO-LiPA Extra test allowed identification of 13 established high-risk HPV types (16, 18, 31, 33, 35, 39, 45, 51, 52, 56, 58, 59, and 68), five known or potential high-risk types (26, 53, 66, 73, and 82), seven low-risk HPV types (6, 11, 40, 43, 44, 54, and 70), additional non-differentiated HPV types, and types with undefined risk (69, 71, and 74).

### Study endpoints

The predefined endpoints were the rates of full HPV vaccination coverage (three doses), the prevalence of HPV 6 and 11 infection, and of genital warts (condylomata acuminata). We reported changes in these prevalence rates according to vaccination status and lifestyle factors (sexual history and smoking history) among participants in the four cohorts.

### Statistical analysis

Vaccination coverage and prevalence rates of HPV 6/11 infection and genital warts are reported for participants aged 19–22 years (cohorts 2 and 4) and 24–27 years (cohorts 1 and 3) at the time of sample collection. Statistical analyses were done to compare similarly aged participants (cohort 1 versus 3 and cohort 2 versus 4) using 2 × 2 contingency tables for the prevalence of HPV 6/11 infection (positive versus negative) and genital warts (no versus yes). The Röhmel-Mansmann unconditional exact test was used to test for difference, with a two-side alpha of 0.05. Statistical analyses were performed using Testimate V.6.5.14.

## Results

Between October 2009 and January 2018, 2456 women were included. Data are available from 2360 women for vaccination status and from 2341 for prevalence of HPV infections and genital warts.

We enrolled 43% of all female residents from cohort 1983/84, 44% in 2009/10 and 47% in 2014/15 (cohort 1988/89) and 25% from cohort 1993/94. At the time of analysis, the study was still ongoing for this cohort (until end of 2020).

### Vaccination coverage

Table 1 shows vaccination coverage for women in each cohort. During the period 10/2009–12/2010, full vaccine coverage rates were 40/659 (6.1%) in participants aged 24–27 years (cohort 1) and 142/600 (23.7%) in those aged 19–22 years (cohort 2). Five years later (11/2014–1/2016), vaccine coverage rates were increased to 135/733 (18.4%) and 177/368 (48.1%) in participants aged 24–27 years (cohort 3) and 19–22 years (cohort 4), respectively.

**Table 1:** HPV vaccination status among the WOLVES cohorts at the time of sample collection. Full vaccination status is defined as 3 doses

Cohort*	Year of analysis	Age at time of sample collection (y)	Full vaccination status, N (%)	
			No	Yes
1	2009/10	24–27	619^a^ (93.9%)	40 (6.1%)
3	2014/15		598^b^ (81.6%)	135 (18.4%)
2	2009/10	19–22	458^c^ (76.3%)	142 (23.7%)
4	2014/15		191^d^ (51.9%)	177 (48.1%)

*Cohort 1 born 1983/84 analyzed in 2009/10; cohort 2 born in 1988/89 analyzed in 2009/10; cohort 3 born 1988/89 analyzed in 2014/15; cohort 4 born 1993/94 analyzed in 2014/15

^a^2 women received 1 dose; ^b^4 women received 1 dose and 7 received 2 doses; ^c^3 women received 1 dose and 9 received 2 doses; ^d^5 women received 1 dose and 8 received 2 doses

### Prevalence of HPV 6 and 11 infection

HPV 6 was the most prevalent of all low-risk HPV type infections (Table 2). There was a statistically significant decrease in the prevalence of HPV 6 infection among women aged 24–27 years (2.1% in cohort 1 versus 0% in cohort 3; *P* < 0.0001) between 2010 and 2015. During this period, there was also a statistically significant decrease in the prevalence of HPV 6 infection among women aged 19–22 years (2.0% in cohort 2 versus 0% in cohort 4; *P* = 0.0056). No cases of HPV 6 infection were seen in the youngest cohort (cohort 4) during follow-up from 2016 to 2018.

**Table 2:** Prevalence of HPV 6 and HPV 11 infection among WOLVES cohorts (2010–2015). Statistical comparisons are shown for cohorts 1 versus 3 and cohorts 2 versus 4

Cohort^a^	N	HPV 6		Rate difference (95% CI)	HPV 11		Rate difference (95% CI	OR
		Negative	Positive		Negative	Positive		
1	659	645 (97.9%)	14 (2.1%)	−0.0212 (− 0.039, − 0.01) *P* < 0.0001	658 (99.9%)	1 (0.1%)	−0.0001 (− 0.0083, 0.0075) *P* = 1.0000	0.000 (not defined; 0.274) *P* < 0.0001
3	714	714 (100%)	0 (0%)		713 (99.9%)	1 (0.1%)		
2	600	588 (98.0%)	12 (2.0%)	−0.02 (−0.0383, − 0.0063) *P* = 0.0056	596 (99.3%)	4 (0.7%)	−0.0067 (− 0.0197, 0.0052) *P* = 0.2753	0.000 (not defined; 0.580) *p* = 0.0048
4	368	368 (100%)	0 (0%)		368 (100%)	0 (0%)		

^a^Cohort 1 born 1983/84 analyzed in 2009/10; cohort 2 born in 1988/89 analyzed in 2009/10; cohort 3 born 1988/89 analyzed in 2014/15; cohort 4 born 1993/94 analyzed in 2014/15

Table 2 shows that the prevalence of HPV 11 infection was low. Among participants aged 24–27 years, the prevalence of HPV 11 infection was 0.1% in cohort 1 and 0.1% in cohort 3, whereas in those aged 19–22 years the rates were 0.7% in cohort 2 and 0% in cohort 4. HPV 11 infection was rare and the decline not obvious. The observed decline in the prevalence of HPV 11 infection was not significant. Furthermore, HPV 11 infection was not linked to clinical disease in the study data analysis.

### Prevalence of genital warts

Between 2010 and 2015, a total of 52 of 2341 participants were diagnosed with genital warts (Table 3). There was a statistically significant decrease in the life-time risk of developing genital warts between participants in cohort 1 and cohort 3 (4.7% versus 1.7%, respectively; *P* = 0.0018). The difference in the life-time risk of developing genital warts between cohorts 2 and 4 (1.3% versus 0.3%) was not statistically significant and there was only one case reported in cohort 4. There was a statistically significant decrease in the overall life-time risk of developing genital warts between participants analyzed in 2010 (cohorts 1 and 2) and those in 2015 (cohorts 3 and 4): 3.1% versus 1.2% respectively; *P* = 0.0022.

**Table 3:** Life-time risk (sum of pre-existing and incident cases) of developing genital warts in WOLVES cohorts (2010–2015). Statistical comparisons are shown for cohorts 1 versus 3, cohorts 2 versus 4, and participants analyzed in 2010 (cohorts 1 + 2) versus those analyzed in 2015 (cohorts 3 + 4)

Cohort^a^	N	Genital warts		Rate difference (95% CI)	OR
		No	Yes		
1	659	628 (95.3%)	31 (4.7%)	0.0302 (0.0109, 0.052)*P* = 0.0018	2.8877 (1.426; 6.226) *p* = 0.0016
3	714	702(98.3%)	12(1.7%)		
2	600	592(98.7%)	8(1.3%)	0.0106(−0.0042, 0.0255)*P* = 0.1598	4.9595 (0.660; 220.656) *p* = 0.1649
4	368	367(99.7%)	1(0.3%)		
1 + 2	1259	1220(96.9%)	39(3.1%)	0.019(0.0068, 0.032) *P* = 0.0022	2.6287 (1.364; 5.395) *p* = 0.0018
3 + 4	1082	1069(98.8%)	13(1.2%)		

^a^Cohort 1 born 1983/84 analyzed in 2009/10; cohort 2 born in 1988/89 analyzed in 2009/10; cohort 3 born 1988/89 analyzed in 2014/15; cohort 4 born 1993/94 analyzed in 2014/15

The numbers of prevalent genital warts diagnosed in the colposcopy clinic in the periods 2010–11 and 2015–16 were as follows: five cases in cohort 1, four cases in cohort 2, three cases in cohort 3, and no case in cohort 4. Out of the nine cases diagnosed in 2010–11, five were classified as typical condylomata acuminata, two as flat condylomata, and two as seborrheic wart-like. The three cases diagnosed in 2015–16 were classified as typical condylomata acuminata (one case) and seborrheic wart-like (two cases). All cases with incident typical condylomata acuminata were associated with detection of HPV 6 infection in cervical samples and in typical condylomata acuminata-tissue.

The cervical smear samples of 5 of 45 patients (11.1%) with genital warts tested positive for HPV 6 or 11 infection, while 17 of 45 (37.8%) tested positive for infection with HPV 6, 11, and high-risk types. The presence of HPV 6 infection in cervical samples directly correlated to the genesis and development of genital warts, while co-infection with HPV 6 and 11 was found in only one case. Overall, 33.3% of participants with genital warts were treated surgically in our dysplasia clinic, 31.1% underwent medical treatment and 35.6% did not receive any treatment.

### Lifestyle factors

Complete sexual behavior and smoking history data in this large population of women showed some differences between cohorts (Table 4). The mean age at first intercourse was 16.9 years in cohort 1, 16.1 years in cohorts 2 and 3, and 16.2 in cohort 4 but this decline was not significant. There were no remarkable differences in numbers of sexual partners.

**Table 4:** Smoking history and sexual experience of study participants^a^

	Cohort 1(1983/84)	Cohort 2(1988/89)	Cohort 3(1993/94)
Number recruited	659	599	368
Smoking history			
Current			
Yes	244 (37%)	221 (37%)	100 (27%)
No	413 (63%)	377 (63%)	268 (73%)
Former			
Yes	163 (25%)	74 (12%)	22 (6%)
No	345 (52%)	392 (65%)	246 (67%)
Age at sexual debut (mean ± SD)	16.9 ± 2.45	16.1 ± 1.67	16,2 ± 1.73
Stable relationship			
Yes	529 (80%)	423 (71%)	264 (72%)
No	123 (28%)	169 (28%)	94 (25%)
Number of sexual partners			
0	8 (1%)	30 (5%)	7 (2%)
1	125 (19%)	126 (21%)	95 (26%)
2–5	335 (51%)	336 (56%)	186 (50%)
> 5	162 (25%)	91 (15%)	44 (12%)

^a^Cohort 1 born 1983/84 analyzed in 2009/10; cohort 2 born in 1988/89 analyzed in 2009/10; cohort 3 born 1988/89 analyzed in 2014/15; cohort 4 born 1993/94 analyzed in 2014/15

This suggest that the reduction of genital warts is unlikely to be explained by changes in sexual behavior or smoking in our population.

Results of univariate testing (Wilcoxon-Mann-Withney-U-Test) and multivariate testing (logistic regression) showed a significant correlation between the number of sexual partners and risk of genital warts in all cohorts (*p* < 0.0001). Young age at first intercourse and currently smoking were not associated with an increased risk for genital warts. We observed a decline in the proportion of current smokers between 2010 and 2015. In 2010, 37% of cohort 2 were smokers compared with 27% in cohort 4 5 years later. The corresponding rates for current smokers were 37% in cohort 1 in 2010 and 27% in cohort 3 in 2015.

## Discussion

The WOLVES study provides the data for this long-term prospective cohort analysis. We focused on a real-life population and measured the impact of HPV vaccination on the prevalence of low-risk HPV and genital warts in Germany only a few years after the introduction of HPV vaccination. In this analysis, we observed a steep drop in the prevalence and incidence of genital warts in women in their mid-twenties from 4.7% among women born in 1983/4 and analyzed in 2010 to 1.7% in those born in 1988/9 and analyzed in 2015. The decrease in genital warts was accompanied by a significant reduction in the prevalence of HPV 6 infection, however, the very low prevalence of HPV 11 had no obvious impact on the development of genital warts. Somewhat unexpectedly, the decrease in genital warts and HPV 6 infection was observed despite the low vaccination coverage in our population (< 50%). It is possible that a single HPV dose may be protective, as suggested by recent publications [14, 15]. In this study, we did not observe a single case of persistent infection with HPV 6, 11, 16, or 18 in women who received one dose of vaccine. Inclusion of all women who received at least 1 dose of vaccine would have resulted in a vaccine coverage rate of 52% in cohort 4 in this analysis.

Previous studies have shown that high levels of HPV vaccine coverage may virtually eliminate genital warts in young women and men (reviewed in [16, 17]). Australia and other countries with a high vaccine coverage already observed the near-extinction of genital warts in adolescents and young adults [18]. Surveillance studies from countries with vaccination programs based on bivalent HPV vaccine and from countries with low vaccine coverage showed a less pronounced decline in the incidence of genital warts. Recent publications showed the early benefit of vaccination such as the decrease in the incidence of anogenital warts in women aged < 22 years and the possible development of herd immunity within population with high vaccination coverage [19].

The results from our study also show a clear correlation between an increase in vaccination coverage and a reduction in the developing of genital warts. In WOLVES, it was notable that a decline of genital warts and complete disappearance of HPV 6 infection was observed in young women with the highest vaccination coverage, i.e. those aged 19–22 years. Nevertheless, the vaccination coverage in our study population was lower than in previously conducted studies, but seems to be high enough to confer herd immunity [20]. A systematic review and meta-analysis of transmission-dynamic models concluded that strong herd effects are expected from vaccinating girls only, even with coverage rates as low as 20% [21]. Other studies in Germany have also suggested the possibility of herd immunity despite low vaccine coverage. Mikolajczyk et al. reported a decrease of the incidence of genital warts in females aged 16 years (47%), 17 years (45%), and 18 years (35%) at the end of 2008, 1 year after the start of public funding of HPV vaccination in young women [22]. Furthermore, Thöne et al. reported a maximum reduction of up to 60% in genital warts among 16- to 20-year-old females and no decrease for corresponding groups outside the range 14–24 years [23]. We interpret our data as a proof that transmission of HPV 6 infection as the causal agent of condylomata acuminata and even of other genital warts is strongly inhibited even in populations with low vaccination coverage, although the exact mechanism of this protection is unknown and the necessary level of vaccination coverage remains uncertain [24].

The major limitation of this analysis is the low number of genital warts reported in our population (52 cases). Our data may also include repeat in-patient treatment of genital warts or visits by the same individual. A number of other factors could potentially have influenced the trends we observed. An increase in the use of self-applied topical treatment of genital warts over time may have contributed to the decline in the surgical treatments seen in our analysis, however, such an effect would be expected in all cohorts and, therefore, the decline observed in our youngest cohort (1993/94) cannot be explained by changes in the topical treatment of genital warts alone. It is possible that some self-reported diagnoses of genital warts were not valid and the true prevalence could be higher than reported in our previous analysis [2]. The rate of underdiagnosed women is probably not related to vaccination status and the low occurrence of genital warts did not have any impact on our analyses*.*

The focus of this paper is on changes in low-risk HPV infection and associated diseases. We did not report data for vulvar intraepithelial neoplasia, which is rarely linked to low-risk HPV infection.

The major strengths of this study are the recruitment of a large general population and the prospective nature of the design, rather than being only a retrospective analysis of existing databases. We recruited women aged 19 to 27 years and to our knowledge, this is the first complete population-based analysis of low-risk HPV infection and genital wart prevalence data from women in this age group in Germany. Between 2015 and 2019, final analyses of all colposcopies and subsequent procedures were done to ensure that the clinical data were robust. The analysis is also strengthened by collection of comprehensive behavioral and lifestyle data, which show that the observed reduction of genital warts is unlikely to be explained by changes in sexual behavior and smoking.

## Conclusions

This analysis of the ongoing WOLVES study adds evidence about the short-term impact of HPV vaccination and shows protection against HPV 6 infection-related genital warts despite low vaccine coverage. Furthermore, an increase in the vaccination coverage rate was associated with decreased prevalence of genital warts in young women. We observed a protective effect with the quadrivalent vaccine on genital HPV 6 infection and an almost fully protective effect on the development of genital warts in the youngest population. We believe that further monitoring of this trend is very important to evaluate the progress of state-sponsored HPV vaccination policies in Germany. Although previous studies and meta-analysis demonstrated the effectiveness of HPV vaccination programs, less is known about the population effectiveness of HPV vaccination and its short-term effects on HPV-related health disparities.

## Data Availability

WOLVES is managed by Klinikum Wolfsburg, the central database is located in the department of gynecology. The contract between the financier, gynecologists in private practice, other partners, and Klinikum Wolfsburg encourages the use of WOLVES data for research and gives all rights for analyses and publications to the head of the department of obstetrics and gynecology as leader of the scientific team.

## Abbreviations

HC2: Hybrid Capture 2
HPV: Human papillomavirus
PCR: Polymerase chain reaction
WOLVES: Wolfsburg HPV epidemiological study
